# Reduced Superficial Capillary Density in Cerebral Infarction Is Inversely Correlated With the NIHSS Score

**DOI:** 10.3389/fnagi.2021.626334

**Published:** 2021-02-25

**Authors:** William Robert Kwapong, Yuying Yan, Zilong Hao, Bo Wu

**Affiliations:** Department of Neurology, West China Hospital, Sichuan University, Chengdu, China

**Keywords:** cerebral infarction, macula capillaries, choriocapillaris, optical coherence tomography angiography, foveal avascular zone

## Abstract

**Purpose**: The retina and the brain share similar neuronal and microvascular features, therein we aimed to assess the structural and microvascular changes in the macula and choriocapillaris (CC) in patients with cerebral infarction when compared with healthy controls using optical coherence tomography angiography (OCTA).

**Methods**: OCTA was used to image and measure the capillary density in the radial peripapillary capillaries (RPC), superficial capillary plexus (SCP), deep capillary plexus (DCP), choriocapillaris (CC), and mean area of the foveal avascular zone (FAZ) in all participants. Twenty-two cerebral infarction patients based on their magnetic resonance imaging (MRI) and 25 healthy controls were included in our study.

**Results**: Density of the RPC (*P* < 0.001), SCP (*P* = 0.001), DCP (*P* < 0.001) and CC (*P* < 0.001) were significantly reduced in cerebral infarction patients when compared with healthy controls, respectively. Retinal thickness measurements (*P* < 0.05) were significantly reduced in cerebral infarction patients when compared with healthy controls. The mean FAZ area was significantly larger (*P* = 0.012) in cerebral infarction patients when compared with healthy controls. National Institute of HealthStroke Scale (NIHSS) inversely correlated with SCP density in cerebral infarction patients (Rho = −0.409, *P* = 0.001). Receiver operating characteristics curve analysis showed that the blood flow of the choriocapillaris had the highest index [area under the receiver operatingcharacteristic (AUROC) = 0.964] to discriminate cerebral infarction patients from the healthy controls.

**Conclusions**: Our study suggests that cerebral microcirculation dysfunction which occurs in cerebral infarction is mirrored in the macula and choroidal microcirculation. OCTA has the potential to non-invasively characterize the macula and choroidal changes in cerebral infarction *in vivo*.

## Introduction

Stroke has been reported to be one of the common causes of death worldwide and is the leading cause of death in China (Zhou et al., 2019). Zhou et al. (2019) reported that with the rapid socioeconomic transition in China, the prevalence of stroke and its risk factors is bound to increase. The pathological changes of stroke involve a decrease in blood flow which leads to insufficient oxygen and cell death in the brain and spinal cord. Unfortunately, the classical clinical manifestation of ischemic stroke, which comprises abrupt unresponsiveness in the face, arm, or leg, particularly on one side of the body, is only apparent after immense blood flow loss in the brain and cell death. Although the neurological discrepancies are typical examples of stroke sequelae, these discrepancies go together with stroke-induced visual dysfunction in over 60% of stroke cases (Rowe, 2017).

Cerebral infarction, a common incidental discovery on magnetic resonance imaging (MRI), has been reported to be linked to ischemic stroke and help foretell its development (Vermeer et al., 2007); it has also been suggested that cerebral infarction is associated with subtle neurological deficits (Vermeer et al., 2007). Nonetheless, the understanding of the pathophysiology of cerebral infarction remains inadequate. Although cerebral microcirculation dysfunction (mainly cerebral ischemia) has been suggested to play a crucial role in the disease pathogenesis of cerebral infarction, gaining more comprehension is challenging due to the scarcity of non-invasive tools that evaluate the cerebral microcirculation quantitatively and consistently.

The retina, an extension of the central nervous system, has been reported to share many neuronal and microvascular features with the brain because of their similar anatomy, physiology, and embryology (Erskine and Herrera, 2014); besides, accumulating reports (Ong et al., 2013, 2015) have suggested that retinal thickness and microvasculature reflect the microstructural and microcirculation of the brain. Recent reports have shown that the retina is a reliable route to access cerebral microcirculation because of their similarities (London et al., 2013). Previous reports (Cooper et al., 2006; Ikram et al., 2006; de Jong et al., 2008; Cheung et al., 2010) using retinal imaging tools such as the fundus camera have suggested that retinal vascular damage such as retinal hemorrhage and microaneurysms are associated with predictors of stroke, cerebral white matter lesions, and other cerebrovascular diseases. Moreover, retinal ischemia, ischemia which affects the retinal cell loss and damage of the optic nerve, has been suggested to co-exist with cerebral infarction and may be responsible for many of the visual impairments that occur in this disease (Osborne et al., 2004; Cooper et al., 2006). Nonetheless, these retinopathy manifestations have been reported to be somewhat late pointers of retinal damage and reflect the late phase of retinal microvascular and structural damage.

Optical coherence tomography angiography (OCTA) is an imaging tool that can non-invasively image the retinal and choroidal microcirculation *in vivo*; OCTA helps clinicians to visualize the retinal and choroidal capillary anastomoses at a high resolution. Advances in the imaging and quantification software of the OCTA have now enabled a more objective quantitative assessment of the capillary network in the retina and choroid, which reflect earlier and subtler changes in the retinal and choroidal microcirculation before the apparent retinal signs emerge.

In this study, we assessed whether OCTA technology has the potential to characterize the retinal structure and retinal and choroidal microvascular changes in patients with cerebral infarction.

## Materials and Methods

### Data Avaliability Statement

The data that support the findings of this study are available on request from the corresponding author. The data are not publicly available due to privacy or ethical restrictions.

### Study Population

**Figure 1:**
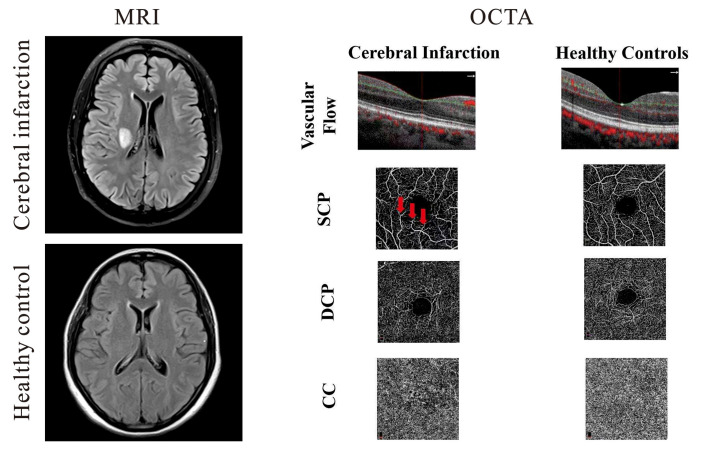
Representative magnetic resonance imaging (MRI) and optical coherence tomography angiography (OCTA) images of cerebral infarction and healthy controls. On the left panel, the upper image shows a T2-weighted MRI of a patient with cerebral infarction beside the right lateral ventricle while the lower right image shows a T2–weighted image of healthy control. Cerebral infarction patients showed reduced vascular flow when compared with healthy controls; macula capillaries were more interrupted in cerebral infarction images when compared with healthy controls. Red arrows represent areas of capillary dropout (middle panel).

Patients presenting to the Neurology Department of West China Hospital, China, were recruited from May 2020 to October 2020. Patients were included if they were Chinese, had cerebral infarction confirmed with magnetic resonance imaging (MRI), could sit adequately, and tolerate retinal imaging using the OCTA. Infarction lesions were confirmed on MR diffusion-weighted imaging consistent with clinical symptoms, as shown in (Figure 1). MR and OCTA imaging were done within 7 days after admission to our hospital. National Institute of Health Stroke Scale (NIHSS) scores were documented. Inclusion criteria of our cerebral infarction patients were as follows: (1) age 20–70 years; and (2) had cerebral infarction which was identified within 1 week of onset. Eligible cerebral infarction cases were matched to controls of the same age group, sex, and race with no self-reported history of stroke or any neurological deficits. Written informed consent was obtained from each participant and approval of our project was obtained from the Ethics Committee of West China Hospital of Sichuan University.

The exclusion criteria of our participants were as follows: (1) diagnosed with diabetic retinopathy or other retinal diseases; (2) Glaucoma; (3) presence of metal fragments in the eyes, brain, or spinal cord; (4) significant media opacities that preclude imaging of the macula; (5) participants with uncertain diagnoses; and (6) participants with a pacemaker or other internal devices; Ultimately, 25 patients were recruited for the ischemic stroke group, and 25 age- and sex-matched controls were enrolled.

After collecting the demographics and clinical information of the participants, ophthalmological examinations such as visual acuity, fundus examination, intraocular pressure examination, and spectral-domain optical coherence tomography (SD-OCT) imaging were completed.

Visual acuity under illumination was completed for each eye using Snellen charts and later converted to the logarithm of the minimum angle of resolution (LogMAR).

### Spectral-Domain Optical Coherence Tomography Imaging

#### Macula Structure

The peripapillary retinal nerve fiber layer (pRNFL) thickness was obtained using the optic nerve head map protocol; the scanning range covered a circle of 3.45 mm in diameter centered on the optic disc. Ganglion cell complex (GCC) thickness was obtained using the GCC scanning protocol which was acquired through scans centered 1 mm temporal to the fovea and covered a square grid (7 mm × 7 mm) on the central macula. Imaging of the structure of the macula was done with the Avanti RTVue-XR (Optovue, Fremont, California, LA, USA, 2017 version).

### Capillaries of the Macula and Choroid

Using the split-spectrum amplitude decorrelation algorithm, OCT angiography (OCTA) images were obtained with Avanti RTVue-XR. The built-in algorithm (version 2017.1.0.151) was equipped with a three-dimensional Projection Artifact Removal (3D PAR) to help reduce all projection artifacts in the deeper layers while keeping their outline and improving the foveal avascular zone (FAZ) area (Liu et al., 2018).

The radial peripapillary capillaries (RPC) network was obtained in scans within a 0.7 mm wide elliptical annular region extending outward from the optic disc boundary, and the vasculature within the internal limiting membrane and the retinal nerve fiber layer (RNFL) were analyzed using the built-in software.

The parafoveal capillary network was obtained through 3 × 3 mm^2^ scans within the annular zone of 0.6 mm–2.5 mm diameter around the foveal center. The superficial capillary plexus (SCP) was 3 μm below the internal limiting membrane to the outer boundary of the inner plexiform layer. The deep capillary plexus (DCP) was described as 15 μm below the INL to 70 μm. The choriocapillaris (CC) was defined as the microvessels within the Bruch’s membrane and the upper boundary of the stroma of the choroid.

The capillary density of the SCP, DCP, and CC was defined as the percentage area occupied by the microvasculature in the analyzed region (3 × 3 mm^2^) and was generated in the whole scan area in all sections of the applied grid according to the Early Treatment Diabetic Retinopathy Study (ETDRS) as previously reported (Yang et al., 2019).

Images with signal quality less than 6 on a scale of 10 were excluded from our data analyses (Lim et al., 2018). Images with motion artifacts seen on the en face images such as irregular patterns of vessels or blurred segmentation of the microvascular plexuses were rejected. Images of good technical quality were included in our data analyses.

### Statistical Analyses

Demographics and clinical information of our participants were described with frequencies (%) and standard deviations as appropriate. Chi-square or independent sample *t*-tests were used to compare the clinical and demographic information between the two groups when appropriate. A generalized estimating equation (GEE) was used to compare the structure, macula microvasculature, and CC between cerebral infarction patients and healthy controls; after, these parameters were then assessed again using GEE while adjusting for inter-eye dependencies, signal quality, and risk factors (gender, hypertension, diabetes, and age). Intraocular pressure of each eye was included as a covariate when analyzing the macula structural thickness between the two groups. The area under the receiver operating characteristic (AUROC) was used to determine the diagnostic accuracy of the analyzed parameters discriminating between cerebral infarction patients and healthy controls. An AUROC of 1.0 indicated perfect discrimination while 0.5 indicated accidental discrimination. *P*-values less than 0.05 were considered statistically significant. SPSS version 22 was used to perform the statistical analyses.

## Results

Twenty-five patients with cerebral infarction were enrolled in this study. Owing to the poor quality of OCTA images of both eyes (movement artifacts, segmentation errors, and signal quality less than 6), three cerebral infarction patients were excluded from our data analyses. Thus, 22 cerebral infarction patients and 25 healthy controls were included in our data analyses. Of our 22 cerebral infarction patients, 15 had subcortical infarction, one had cortical and subcortical infarction, and six had brain stem infarction as shown in (Table 1). There were no significant differences in age, gender, axial length, and IOP between the two groups. Significant differences (*P* < 0.05) were shown in the risk factors such as hypertension and diabetes between the two groups (Table 1). Cerebral infarction patients showed significantly reduced visual acuity when compared with healthy controls (*P* < 0.001).

**Table 1:** Demographics and clinical data.

	Cerebral infarction	Healthy controls	*P*-value
Number	22	25	
Gender (F:M)	2:20	3:22	0.891
Age (years)	54.35 (11.42)	54.41 (9.97)	0.985
BMI	24.23 (2.54)	23.95 (2.16)	0.793
Hypertension (number)	9	3	<0.05
Diabetes (number)	5	1	<0.05
Hyperlipidemia	5	0	<0.05
Smokers (number)	14	9	0.083
Alcohol (Smokers)	8	6	0.311
Location of infarction			
Subcortical *n* (%)	15 (68.2)		
Cortical and subcortical *n* (%)	1 (4.5)		
Brain stem	6 (27.3)		
NIHSS score	2.53 (2.32)	0	
Ophthalmology exam
IOP (mmHg)	15.28 (2.48)	14.96 (2.32)	0.812
AL (mm)	23.16 (1.19)	23.45 (0.78)	0.958
Visual acuity, Snellen chart	0.74 (0.31)	1.10 (0.17)	<0.001
Visual acuity, LogMAR	0.21 (0.23)	−0.05 (0.07)	<0.001

*Data were expressed as mean (standard deviations) or percentages. Chi-square or independent sample *t*-tests were used in comparing characteristics between groups. Text in blue font indicates location of infarct*.

Cross-sectional OCTA images of the macula with visible flow signal through the center of the fovea and in the circumferential portion of the macula were significantly different between the two groups; cerebral infarction patients showed significantly reduced vascular flow when compared with the healthy controls (Figure 1). On the en face angiograms, cerebral infarction patients showed narrower and interrupted capillaries when compared with healthy controls (Figure 1, middle column). Signal quality was significantly different (*P* = 0.03) between patients with cerebral infarction (8.33 ± 1.17) and healthy controls (9.03 ± 0.72).

## Macula Thickness Between Ischemic Stroke and Healthy Controls

pRNFL thickness (*P* = 0.008), RNFL thickness (*P* = 0.02, Table 2) and GCC thickness (*P* = 0.018, Table 2) were significantly reduced in cerebral infarction patients when compared with healthy controls, respectively.

**Table 2:** Comparison of the macula and choriocapillaris between cerebral infarction and healthy controls.

	Cerebral infarction	Healthy controls	*P*-value	*P*-value
RPC (%)	49.02 (3.0)	53.01 (2.54)	<0.001	*<0.001*
pRNFL (μm)	112.41 (11.89)	120.71 (8.68)	<0.003	*<0.008*
RNFL (μm)	104.02 (8.63)	108.94 (7.65)	<0.025	*<0.020*
GCC (μm)	98.43 (6.73)	101.74 (5.05)	<0.014	*<0.018*
SCP (%)	45.93 (3.69)	48.22 (1.43)	<0.002	*<0.001*
DCP (%)	48.50 (3.48)	52.54 (2.27)	<0.001	*<0.001*
FAZ (mm^2^)	0.30 (0.10)	0.22 (0.63)	<0.001	*<0.001*
Choriocapillaris (%)	61.61 (4.58)	68.75 (1.11)	<0.001	*<0.001*

*Data were adjusted for intereye dependencies, hypertension, diabetes, age, gender, and signal quality. P-value: adjusted for signal quality. P-value (italics): adjusted for signal quality and risk factor. Text in blue font indicates the significance of OCT changes with (last column, P value indented) and without (first P value column, P value not indented) adjusting for risk factors*.

The mean FAZ area was significantly larger in cerebral infarction patients when compared with healthy controls (*P* < 0.001, Table 2).

## The Capillary Density Between Ischemic Stroke and Healthy Controls

RPC density (*P* < 0.001, Table 2), SCP density (*P* = 0.001, Table 2) and DCP density (*P* < 0.001, Table 2) were significantly reduced in cerebral infarction patients when compared with healthy controls, respectively. The density in the choriocapillaris was significantly reduced in cerebral infarction patients when compared with healthy controls (*P* < 0.001, Table 2).

Mean area of the FAZ in cerebral infarction patients inversely correlated with the DCP density (Rho = −0.288, *P* = 0.001) and blood flow in the choriocapillaris (Rho = −0.264, *P* = 0.002), respectively.

NIHSS also inversely correlated with the SCP density in patients with cerebral infarction (Rho = −0.409, *P* = 0.001).

**Figure 2:**
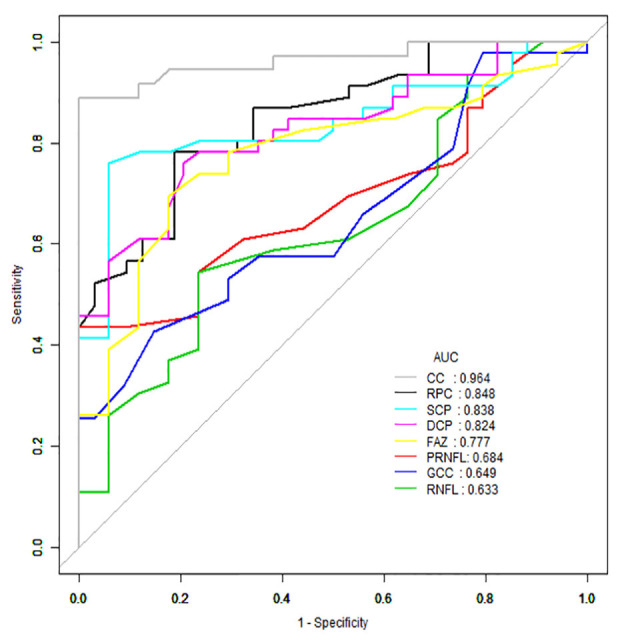
Receiver operating characteristic (ROC) curve analysis of the macula parameters and choriocapillaris (CC). The highest area under the curve (AUC) was the choriocapillaris blood flow (0.964, 95% CI = 0.889–0.994).

AUROC analysis was used to reflect the diagnostic accuracy for each OCTA parameter to provide a distinction between cerebral infarction patients and healthy controls. The highest AUROC result was the choriocapillaris blood flow (0.964, 95% CI = 0.889–0.994; Figure 2, Table 3).

**Table 3:** ROC curve analysis of the macula and choriocapillaris of cerebral infarction and healthy controls.

	AUC	Sensitivity	Specificity
RPC (%)	0.848	78.26	81.25
pRNFL (μm)	0.684	43.48	100
RNFL (μm)	0.633	54.35	76.47
GCC (μm)	0.649	42.55	85.29
SCP (%)	0.838	76.09	94.12
DCP (%)	0.824	76.09	79.41
FAZ (mm^2^)	0.777	69.57	82.35
Choriocapillaris (%)	0.964	88.89	100

## Discussion

In our observational cross-sectional study, we assessed the retinal, structural, and microvascular changes in patients with cerebral infarction. Our current study compared the sub-retinal thicknesses and microvascular densities in the two macula capillary plexuses (superficial and deep), capillaries around the optic nerve, and choriocapillaris. When compared with healthy controls, patients with cerebral infarction showed significantly thinner sub-retina thickness (pRNFL, RNFL, and GCC) and significantly reduced RPC, SCP, DCP, and CC densities. Our report adds to the notion that gradual modifications occur in the retinal structure and microvasculature and choriocapillaris in patients with cerebral infarction. Taken together with accumulating reports, our current study shows that changes in retinal structure and microvasculature and choriocapillaris using the OCTA may potentially be used in monitoring the retinal changes in cerebral infarction.

Our findings on the sub-retinal layer thinning (pRNFL, RNFL, GCC) in cerebral infarction patients when compared with healthy controls are congruent with previous reports on cerebral vascular diseases (Kim et al., 2011; Moss, 2015). The pRNFL, RNFL, and GCC contains axons, cell bodies, and dendrites, thus a significant thinning of these layers reflects a significant neuronal and axonal loss which is congruent with previous cerebral reports (Brundel et al., 2012; Smith et al., 2012).

The growing number of reports (Mitchell et al., 2005; Baker et al., 2008; Wang et al., 2011; Kawasaki et al., 2012) providing evidence of significantly altered retinal microvascular structures in cerebral infarction indicates the importance of assessing the retinal microvasculature *in vivo*. This may shed light on the role of the microcirculatory networks and the development of the disease. Moreover, currently available and widely accepted imaging modalities such as magnetic resonance angiography are expensive, lack specificity, and have their contraindications. Therein, the use of retinal imaging may help in understanding the pathologic mechanism underlying the disease cascade and can be complementary to these neuroimaging tools. The OCTA is an imaging tool that helps in the acquisition of high-resolution *in vivo* cross-sectional images and assessable measurements of the optic disc, macula, and choriocapillaris, which have been reported to be associated with the cerebral structures (microvasculature and microstructure). It has been suggested to be particularly useful in many neurodegenerative diseases and a useful tool in examining the course of development (Wylęgała, 2018; Pujari et al., 2020). The usefulness and potential of OCTA to determine capillary-level detail make it an important imaging modality that can provide in-depth information regarding impaired perfusion. It allows clinicians to assess the severity of ischemia (Pujari et al., 2020) with much more precision which may be beneficial in understanding the pathophysiology of cerebral infarction.

Our data observed a significant dropout of macula microvasculature (SCP and DCP) in cerebral infarction patients when compared with healthy controls. Previous cerebral imaging reports have suggested that vascular dysfunction leads to cerebral hypoperfusion during the development of ischemic cerebrovascular lesions (Kunz and Iadecola, 2009; Hu et al., 2017). Moreover, it has been suggested that before cerebral infarction, most patients may have had combinations of large-vessel stenosis, small vessel disease, and a propensity to form either cardiac emboli or arterial thrombi with atherosclerotic lesions. This may then result in neural cell loss/death and a significant reduction in cerebral microcirculation dropout. With the association between cerebral microcirculation and retina microcirculation, the retinal microcirculation may be reflective of the pathophysiological cascade in cerebral infarction. Ideally, we found that the capillary damage in the deep plexus was more severe than in the superficial plexus. The deep capillary plexus which is located in the deepest part of the inner retina is reported to consist solely of capillaries, unlike the superficial plexus which consists of capillaries, arterioles, and venules. The capillaries in the DCP are thinner and have a smaller cross-sectional area making them sensitive to any disease that affects the retina. However, our study is observational and did not explore the contributory mechanisms.

Our report also showed that patients with cerebral infarction have a significantly reduced RPC density when compared with healthy controls. Our report is in agreement with a previous study that showed reduced vascular density around the optic nerve using fundus photography (Sprodhuber et al., 2018). Although vessels imaged and measured from fundus photography are considerably larger in diameter than those obtained from the OCTA (Forrester et al., 1996), our report echoes the aforementioned report.

A novel finding in our study was the significant density dropout in the choriocapillaris of cerebral infarction patients when compared with healthy controls. It has been suggested that the occurrence of cerebral infarction is associated with aging and atherosclerosis of the vessels (Lernfelt et al., 2002; Liu et al., 2017), which create an ischemic environment, therein affecting the structure and microvasculature of the choroid. Significant dropout in the choriocapillaris remained after adjusting for age and other risk factors suggesting that changes in the choriocapillaris due to the disease mechanism. Future studies will be needed to validate our hypothesis.

The FAZ is located in the inner retina (mostly in the deep capillary plexus region) and it is made up of photoreceptor cells and bound by interconnected capillary anastomoses (Tick et al., 2011; Bates et al., 2018). Our current study showed that the FAZ area was larger in cerebral infarction patients when compared with healthy participants and observed significant inverse associations between the FAZ area and DCP and CC, respectively. The FAZ enlargement in patients with cerebral infarction may be due to retinal degeneration as seen in our current report and previous reports. Another possible explanation for the FAZ enlargement may be the significant dropout in the microvascular as shown in our current report as well.

An inverse correlation between the NIHSS score and density in the SCP was another new finding in our report. NIHSS, an important indicator to assess the severity of a stroke, has been reported to reflect the severity of neurological impairment (Lyden, 2017). An inverse correlation between NIHSS score and the density in the SCP of patients with cerebral infarction indicates that the higher the NIHSS score, the lower the density in the SCP and* vice versa*. Although this is the first report to highlight the correlation between these two parameters, future studies are needed to validate our speculation.

The significant difference between healthy controls and cerebral infarction provides a potentially clinically useful screening indicator for detecting the presence of cerebral infarction. Despite a promising area under the curve (AUC) in the ROC analysis with a lower 95% CI of greater than 0.5, studies using a longitudinal approach may be needed to validate our findings. Nonetheless, our results suggest that measurement of the blood flow in the choriocapillaris using OCTA can be used to identify cerebral infarction patients and may constitute an early indicator to help screen for patients with cerebral infarction. Identifying the significant reduction of the choriocapillaris in cerebral infarction patients before the occurrence of the apparent retinopathy signs seen on fundus photography may help clinicians to apply earlier implementation of treatment and may be useful to predict the progression of the disease as well.

A major limitation in our current study is the small sample size and inclusion of only Chinese participants. Second, individuals were excluded with a known vascular disease from our study; therein, we could not determine whether these results were translatable to individuals with retinal and choriocapillaris changes due to other causes. Another limitation is the statistical difference in the clinical information such as the number of hypertensives and diabetics included in our data. The OCTA technique requires participants to focus and cooperate, which makes some of the images obtained unsuitable for analysis.

## Conclusions

Notwithstanding our limitations, we showed that patients with cerebral infarction can be imaged and detected by the OCTA which is a quick, non-invasive, inexpensive tool based on the significant reduction of blood flow in the choriocapillaris. Our report showed that patients with cerebral infarction have significantly reduced macular microvascular and choriocapillaris densities when compared with healthy controls. We also showed that reduced SCP density is inversely correlated with NIHSS score in cerebral infarction patients. Our findings suggest that these microvascular changes in the retina and choriocapillaris may reflect the cerebral microcirculation in cerebral infarction and demonstrate the potential of OCTA for early screening of cerebral infarction patients.

## Data Availability Statement

The raw data supporting the conclusions of this article will be made available by the authors, without undue reservation.

## Ethics Statement

The studies involving human participants were reviewed and approved by West China Hospital Ethics Committee. The patients/participants provided their written informed consent to participate in this study.

## Author Contributions

All authors listed have made a substantial, direct and intellectual contribution to the work, and approved it for publication.

## Conflict of Interest

The authors declare that the research was conducted in the absence of any commercial or financial relationships that could be construed as a potential conflict of interest.
